# Resection of the Scapula

**Published:** 1849-02

**Authors:** 


*Resection of the Scapula.*—W. ——, aged 32, was admitted into King's College Hospital, January 13th, 1847. He had suffered from disease of the right shoulder-joint for seven years, when the extremity was amputated at the articulation, at Fort Pitt Hospital, and a part of the glenoid cavity was at the same time removed. He recovered, but became the subject of abscess on the front of the chest about twelve months afterwards. On admission into King's College Hospital, the whole shoulder was enlarged, and the soft tissues hypertrophied ; a great many fistulous openings existed in the pectoral region, and over the clavicle, which discharged copiously. Having determined on the propriety of removing the scapula entire, Mr. Fergusson proceeded to operate on the 6th of Feb., 1847. The patient was put under the influence of æther, and the clavicle was first exposed, and di vided about two inches from its acromial extremity, with a saw, another incision extended along the spine of the scapula ; and a third in the course of the old cicatrix. Some further dissection, and a division of the attaching muscles, enabled the operator to complete the excision, the subclavian artery being the while compressed over the first rib. The axillary and other arteries were tied, and the wound was closed with stitches. The patient's recovery was satisfactory, and unattended by anything deserving particular notice. On May 5th he was sent into the country, and a recent account reports him to be well and fat, though still occasionally troubled with small abscesses on the breast. After maceration, the bone exhibited a hypertrophied condition ; the remaining portion of the glenoid cavity was carious, and its margin was surrouudnd by a mass of new ossific matter.—*Prov. Med. and Surg. Journ.*

